# Extensive Lower Extremity Venous Thrombosis and Phlegmasia From Heparin-Induced Thrombocytopenia Following Percutaneous Coronary Intervention (PCI) Successfully Managed With Limb-Salvage Surgery

**DOI:** 10.7759/cureus.101410

**Published:** 2026-01-13

**Authors:** Carlos Diaz-Sepulveda, Sebastian Castañer-Colberg, Gabriel Pereira-Torrellas, Rafael Santini-Dominguez

**Affiliations:** 1 Surgery, Centro Médico Episcopal San Lucas, Ponce, PRI; 2 General Surgery, Centro Médico Episcopal San Lucas, Ponce, PRI; 3 Vascular Surgery, Centro Médico Episcopal San Lucas, Ponce, PRI; 4 General Surgery, St Luke's Episcopal Medical Center, Ponce, PRI

**Keywords:** argatroban, below-knee amputation, deep vein thrombosis, heparin-induced thrombocytopenia, hypercoagulable state, non-heparin anticoagulation, percutaneous coronary intervention, phlegmasia cerulea dolens, vascular surgery, venous gangrene

## Abstract

We report the case of a 64-year-old woman recently treated with unfractionated heparin during percutaneous coronary intervention who developed progressive left lower extremity edema after hospital discharge. Over the next several days, she experienced worsening swelling, sensory loss, and subsequent cyanosis. She presented to the emergency department with marked limb swelling, mottling, leukocytosis, thrombocytopenia, and elevated D-dimer. Duplex ultrasonography revealed extensive iliofemoral and infrapopliteal thrombosis. Given recent heparin exposure and new thrombocytopenia, her 4Ts score indicated intermediate probability for heparin-induced thrombocytopenia (HIT), prompting immediate discontinuation of heparin and initiation of argatroban. PF4/heparin enzyme-linked immunosorbent assay (ELISA) and serotonin release assay later confirmed HIT. Due to worsening venous congestion consistent with evolving phlegmasia cerulea dolens (PCD), she underwent emergent fasciotomy and open thrombectomy, resulting in restored venous outflow and limb reperfusion. Although she subsequently required a left below-knee amputation due to irreversible distal ischemia, preservation of the knee joint provided a markedly better functional prognosis than an above-knee amputation. She remained hemodynamically stable, achieved platelet recovery on non-heparin anticoagulation, and survived a condition historically associated with high mortality. This case highlights the potential for HIT to present with limb-threatening thrombosis after hospital discharge and the importance of early recognition, appropriate anticoagulation, and timely surgical intervention in optimizing limb and patient outcomes.

## Introduction

Heparin-induced thrombocytopenia (HIT) is a potentially life-threatening, immune-mediated adverse reaction to heparin characterized by paradoxical thrombocytopenia and a marked prothrombotic state [1-4]. The syndrome results from IgG antibodies directed against platelet factor 4 (PF4)/heparin complexes that activate platelets through FcγIIa receptors, triggering thrombin generation and amplification of coagulation pathways [1-4]. Although thrombocytopenia is a defining feature of HIT, bleeding is uncommon, and the principal clinical danger arises from a profound prothrombotic state leading to venous, arterial, and microvascular thrombosis [1,2]. Without appropriate management, thrombotic complications develop in up to 50% of affected patients, often with devastating consequences [5,6].

Heparin-induced thrombocytopenia most commonly presents with a decline in platelet count beginning five to 10 days after exposure to unfractionated or low-molecular-weight heparin [7]. This phenomenon is particularly relevant in patients with cardiovascular disease receiving heparin during interventional procedures. Recognition of HIT relies on using clinical probability tools, like the 4Ts score, with additional confirmatory tests, like the HIT-antibody test (PF4 enzyme-linked immunosorbent assay (ELISA)) and the serotonin release assay (SRA) [8,9]. Current guidelines emphasize immediate cessation of heparin and prompt initiation of non-heparin anticoagulation when HIT is suspected with at least intermediate pretest probability [2,5,8].

Phlegmasia cerulea dolens (PCD) represents the most severe clinical expression of deep venous thrombosis. Characterized by massive venous obstruction, intense edema, compromised arterial inflow, and cyanosis, PCD may rapidly progress to venous gangrene, with amputation rates approaching 20-50% and mortality as high as 25% [10,11]. Cases of HIT-associated PCD are rarely reported but consistently demonstrate a fulminant course and a high risk of major limb amputation [10,11].

Percutaneous coronary intervention (PCI) routinely requires systemic anticoagulation, most commonly with unfractionated heparin. Despite this widespread exposure, heparin-induced thrombocytopenia remains an uncommon complication in the PCI population, with reported incidence rates of approximately 0.3% [12].

The present case is academically significant for several reasons. First, heparin-induced thrombocytopenia following PCI is rare. Second, the accelerated progression of HIT resulting in PCD usually requires above-the-knee amputations [10,11]. In this patient, the preservation of the knee joint provided markedly better long-term functional outcomes than an above-knee amputation [13,14]. Finally, the case highlights that, despite the historically high mortality associated with PCD [10,11], early recognition of HIT, rapid initiation of argatroban, and timely surgical intervention contributed to patient survival. Together, these features highlight the importance of maintaining a high index of suspicion for HIT in any patient with new thrombocytopenia and rapidly progressive thrombosis after recent heparin exposure, regardless of the setting in which symptoms arise.

## Case presentation

A 64-year-old woman with hypertension, diabetes mellitus, dyslipidemia, obesity, and coronary artery disease was hospitalized for non-ST-elevation myocardial infarction. Initially, she underwent a diagnostic left heart catheterization, followed by PCI with stent placement to the right coronary artery the following day; unfractionated heparin was administered during both procedures. Labs were ordered post-PCI day 1, and her platelet levels were within her baseline. No additional labs were ordered during this admission. She was discharged home on post-PCI day 7. 

**Figure 1:**
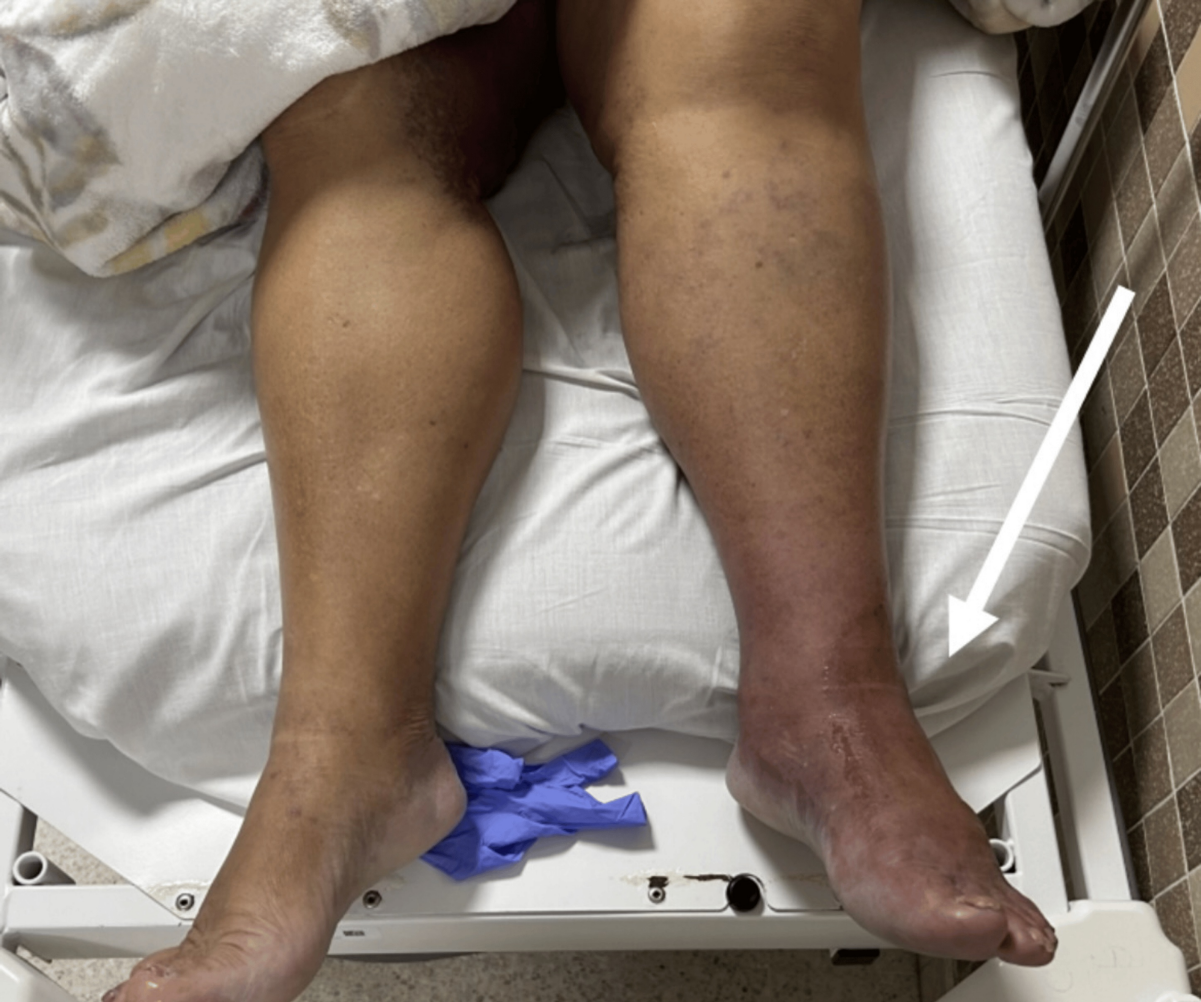
Gross appearance of the left lower extremity on presentation Clinical photograph of the left lower extremity demonstrating marked edema, cyanosis, and mottling.

Following discharge, she experienced progressive swelling of the left lower extremity. By post-PCI day 9, she noted worsening pain with new sensory loss. On post-PCI day 10, she observed bluish discoloration of the leg. Despite escalating symptoms, she remained at home until post-PCI day 11, when she presented to the emergency department with marked swelling, cyanosis, and difficulty bearing weight, consistent with severe venous congestion (Figure 1). 

**Figure 2:**
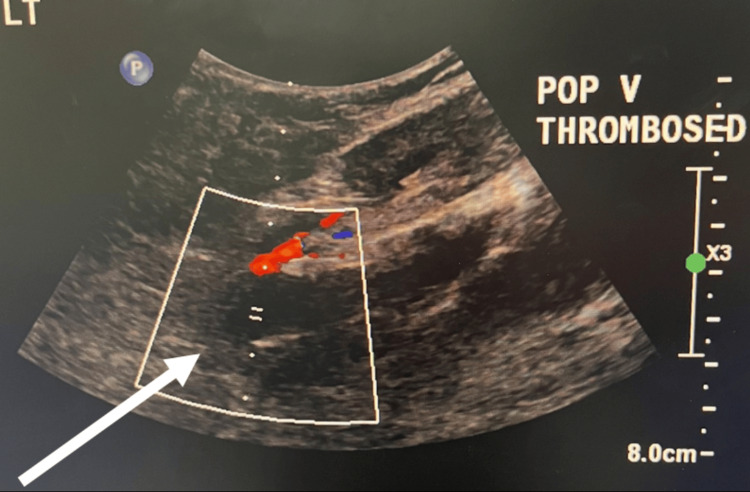
Duplex ultrasonography of the left popliteal vein Duplex ultrasonography demonstrating intraluminal thrombus within the left popliteal vein (arrow) with markedly reduced color Doppler flow.

On arrival, her left lower extremity was markedly edematous, tense, and mottled, with delayed capillary refill and decreased sensation. Distal pulses were detectable only by Doppler. Laboratory evaluation revealed leukocytosis, new thrombocytopenia compared with her pre-procedural baseline, and markedly elevated D-dimer levels (Table 1). Duplex ultrasonography demonstrated extensive occlusive thrombosis involving the left popliteal, posterior tibial, and peroneal veins (Figure 2).

**Table 1:** Initial laboratory investigations on presentation FEU: fibrinogen equivalent units

Test	Value	Unit	Reference Range
White blood cells	15.73	×10³/µL	4.0-11.0
Platelets (baseline)	155	×10³/µL	150-450
Platelets (presentation)	27	×10³/µL	150-450
D-dimer	35.2	mg/L FEU	<0.50

Given her recent unfractionated heparin exposure, new thrombocytopenia, and extensive thrombosis, her 4Ts score was calculated and indicated intermediate probability for HIT. Argatroban infusion was initiated, and the patient was admitted to the Surgery Intensive Care Unit (SICU) and optimized for surgery. 

**Figure 3:**
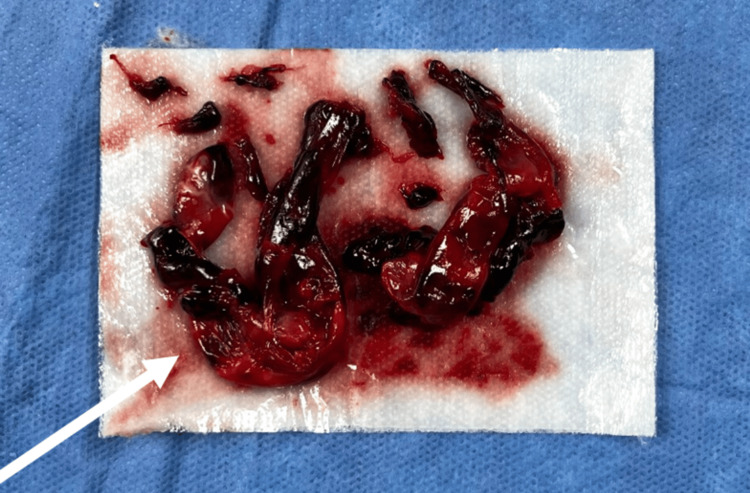
Intraoperative thrombus removed during popliteal vein thrombectomy Gross appearance of thrombotic material removed from the left popliteal vein during open thrombectomy (arrow).

Because of rapid progression toward PCD with concern for limb-threatening venous gangrene, vascular surgery performed an emergent four-compartment fasciotomy and popliteal vein open thrombectomy. A large organized thrombus was removed during open popliteal vein thrombectomy (Figure 3), and venous outflow was restored with immediate reperfusion of the limb.

Postoperatively, the patient was transferred to the SICU for continued argatroban therapy and close monitoring. Hematology services were consulted, who ordered the PF4/heparin ELISA and the SRA confirmatory tests, and both returned positive (Table 2), establishing the official diagnosis of HIT. Despite initial stabilization and transfer to the general ward, the patient developed irreversible distal ischemia despite restoration of proximal venous outflow. This ischemia manifested as cyanotic toes, bullae around the ankle, decreased toe sensation, and markedly reduced toe range of motion. Given the non-viability of the distal limb, she ultimately required a left below-knee amputation. Following the procedure, she stabilized as perfusion to the remaining limb segments improved and was later discharged with long-term non-heparin anticoagulation.

**Table 2:** Heparin-induced thrombocytopenia confirmatory testing ELISA: enzyme-linked immunosorbent assay; OD: optical density; PF4: platelet factor 4; SRA: serotonin release assay

Test	Value	Unit	Reference Range
PF4/heparin ELISA	2.745 (positive)	OD	<0.40
SRA, low-dose heparin	92 (positive)	%	<20%

## Discussion

This case illustrates a rare but devastating manifestation of HIT presenting as PCD after hospital discharge. While HIT is most often recognized during inpatient care, this case reinforces that clinically significant thrombosis may evolve entirely in the outpatient setting, even after an initially uncomplicated hospitalization. The patient’s delayed presentation underscores a critical diagnostic vulnerability, as early symptoms of HIT-related thrombosis may be subtle or misattributed once patients leave the monitored hospital environment. Several features of this case warrant highlighting. First, the development of heparin-induced thrombocytopenia after PCI is rare; progression to PCD, as observed in this case, is exceedingly uncommon and highlights the importance of early recognition and timely intervention to prevent catastrophic outcomes. Second, reported instances of HIT-associated PCD highlight its fulminant progression to venous gangrene, major limb amputation, and death despite intervention, underscoring the lethality of this condition. This reflects the massive venous occlusion and rapid ischemic progression characteristic of PCD [15,16]. Compared with many previously reported cases, the patient's outcome was relatively favorable. Early clinical suspicion based on timing, thrombosis, and platelet trends prompted discontinuation of heparin and initiation of argatroban before confirmatory testing results were available. Rapid involvement of vascular surgery and emergent fasciotomy with venous thrombectomy restored proximal venous outflow and limited the extent of ischemic injury. Although distal tissue necrosis ultimately required a below-knee amputation, preservation of the knee joint represents a meaningful functional outcome, as below-knee amputation is consistently associated with improved mobility, prosthetic use, and rehabilitation potential compared with above-knee amputation.

## Conclusions

In conclusion, this patient survived a condition historically associated with high mortality. Her course illustrates that early recognition of HIT, prompt initiation of non-heparin anticoagulation, and timely surgical intervention can alter the natural history of HIT-associated PCD, improving both survival and limb-related outcomes even when complete limb salvage is not possible. This case reinforces the need for continued vigilance for HIT after hospital discharge, particularly in patients exposed to unfractionated heparin during cardiovascular interventions, and highlights the importance of multidisciplinary management when limb-threatening thrombosis is suspected.
